# Factors associated with hospital outcomes of patients with penetrating craniocerebral injuries in armed conflict areas of the Democratic Republic of the Congo: a retrospective series

**DOI:** 10.1186/s12873-021-00504-5

**Published:** 2021-10-02

**Authors:** Paterne Safari Mudekereza, Gauthier Bahizire Murhula, Charles Kachungunu, Amani Mudekereza, Fabrice Cikomola, Leon-Emmanuel Mukengeshai Mubenga, Patrick Birindwa Balungwe, Paul Munguakonkwa Budema, Christian Molima, Erick Namegabe Mugabo, Hervé Monka Lekuya

**Affiliations:** 1 Faculté de Médecine, Université Catholique de Bukavu, Bukavu, Democratic Republic of the Congo; 2 Hôpital Provincial Général de Référence de Bukavu, Bukavu, Democratic Republic of the Congo; 3 Société Congolaise de Neurochirurgie (SCNC), Kinshasa, Democratic Republic of the Congo; 4 Ecole Régionale de Santé Publique, Université Catholique de Bukavu, Bukavu, Democratic Republic of the Congo; 5 Department of Surgery, CHS, Makerere University, P.O. Box 7072, Kampala, Uganda

**Keywords:** Penetrating craniocerebral injury, Intracerebral hemorrhage, Hemodynamic instability, Hospital outcomes, Glasgow outcome score

## Abstract

**Introduction:**

Penetrating craniocerebral injuries (PCCI) are types of open head injuries caused by sharp objects or missiles, resulting in communication between the cranial cavity and the external environment. This condition is deemed to be more prevalent in armed conflict regions where both civilians and military are frequently assaulted on the head, but paradoxically their hospital outcomes are under-reported. We aimed to identify factors associated with poor hospital outcomes of patients with PCCI.

**Methods:**

This was a retrospective series of patients admitted at the Regional Hospital of Bukavu, DRC, from 2010 to 2020. We retrieved medical records of patients with PCCI operated in the surgical departments. A multivariate logistic regression model was performed to find associations between patients’ admission clinico-radiological parameters and hospital outcomes. Poor outcome was defined as a Glasgow Outcomes Score below 4.

**Results:**

The prevalence of PCCI was 9.1% (91/858 cases) among admitted TBI patients. More than one-third (36.2%) of patients were admitted with GCS < 13, and 40.6% of them were unstable hemodynamic. Hemiplegia was found in 23.1% on admission. Eight patients had an intracerebral hemorrhage. Among the 69 operated patients, complications, mainly infectious, occurred in half (50.7%) of patients. Poor hospital outcomes were observed in 30.4% and associated with an admission GCS < 13, hemodynamic instability, intracerebral hemorrhage, and hemiplegia (*p* < 0.05).

**Conclusion:**

The hospital poor outcomes are observed when patients present with hemodynamic instability, an admission GCS < 13, intracerebral hemorrhage, and hemiplegia. There is a need for optimizing the initial care of patients with PCCI in armed conflict regions.

## Introduction

Penetrating craniocerebral injury (PCCI) are types of open head injuries caused by sharp objects or missiles, resulting in communication between the cranial cavity and the external environment; frequently the sharp object or missile does not exit and remains stuck [1, 2]. This condition is deemed to be more prevalent in armed conflict regions where both civilians and military are frequently assaulted on the head with a missile or sharp objects [1, 2]. The outcomes of PCCI are reported to be poor even in high-income countries (HICs), mainly focusing on ballistic injuries [3–5]. They are influenced by factors like some demographics factors (age), some clinical factors (low GCS, non-reactive pupils), and some radiological factors (bi-hemispheric lesion, basal cistern opening) [3, 5].

Knowing these associated factors of outcomes could help clinicians to take further decisions and adapt the management strategy of PCCIs. Although the outcomes of PCCI are understudied in low and middle-income countries (LMICs), they are not spared from this public health issue. Some studies have shown that PCCI have favorable outcomes and a low mortality rate in LMICs because many victims of PCCIs died at the injury site and those who rarely arrive at the hospital have a high survival potential [6, 7]. Few studies conducted in armed conflicts context in Africa and other LMICs have demonstrated that patients with GCS < 8 and bi-hemispheric lesions have more fatal outcomes compared to others [8, 9]. But outcomes of PCCI in armed conflicts region by taking into account all the penetrating agents (gunshot and non-missiles) have not been established to our knowledge.

Indeed, the Eastern region of the Democratic Republic of the Congo (DRC) has been a war site of recurrent rebellions due to political instability for more than 20 years. Both civilian and military victims often sustain severe traumatic brain injury (TBI), especially the PCCI by different mechanisms like gunshot, and machetes cut on the head. There are relatively few specialized trauma centers in that armed conflict region of the country to manage severe TBI. The surgical workforce is very limited, and accessible only in the regional referral hospitals. The current trauma guidelines are not yet properly standardized, and alternative strategies to improve the care of PCCI could focus on the predictor parameters of the outcomes of management. Moreover, there still a lack of studies on the PCCI outcomes in LMICs [10]. This study aims to highlight the factors associated with the outcomes of management of PCCI at discharge from the surgical units in order to guide clinicians in decision-making and to improve pre-hospital trauma care such as emergency medical mobile services.

## Methods

### Study design and setting

This was a retrospective study conducted at the Surgical Department of the Regional Referral Hospital of Bukavu (HPGRB), Bukavu town, Sud-Kivu, DRC. This referral hospital receives patients from the entire population in the armed zones of the Eastern region of the Democratic Republic of the Congo.

### Population and variables

We retrieved medical records of all age group patients admitted at HPGRB with the documented diagnosis of PCCI, confirmed by clinical assessment and radiological examinations from January 2010 to April 2020. Patients with incomplete medical records were excluded from our study. We collected data using a data collection tool with different predictor and outcome variables such as patients’ demographics, mechanisms of injury, clinico-radiological findings, and hospital outcomes. The main outcome of the study was the Glasgow outcomes score (GOS) at discharge and divided into 2 groups: favorable outcomes when GOS is 4 to 5, and poor outcomes below 4. Data were entered into an Excel sheet form that was saved on Google Drive for data backup. Data analysis was done using Statistical Data Analysis (STATA^R^) v15.1. Continuous variables were described in terms of medians (interquartile range) and mean (+/− standard deviation). Categorical variables were described by frequencies and percentages. Chi-square test and Fisher’s exact tests (where appropriated) were used to compare proportions. A multivariate logistic regression model was performed to identify patients’ factors and clinico-radiological parameters (on admission) of poor outcomes. The odds ratios of the statistically significant factors were used to determine the association between patients and PCCI characteristics and the outcomes. Statistical significance was assessed by *p* < 0.05.

### Ethical consideration

This study received the approval of the Ethical Committee of the Catholic University of Bukavu. This study respects also all Good Clinical Practice used in clinical research in accordance with Helsinki Declaration [11].

## Results

### Demographics and injury factors

There was a total of 91 patients with PCCI. Only 69 patients had relatively satisfactory medical records. The prevalence of PCCI was 9.1% (91 of 858 cases of moderate to severe traumatic brain injury). The mean age of our study population was 26.5 years (+/− 16.2); among the 69 patients, more than half (55.1%) were aged from 16 to 45 years, whereas 30.4% were aged between 0 and 15 years and 14.5% between 46 and 70 years. The male-female sex ratio was 3. About 68% of those patients were war victims, and half of them were injured by gunshot and explosive, whereas 31.8% were victims of other causes (machete cut, fall, etc.). A large number of them (42/69) patients were coming from rural areas.

### Clinical presentation and imaging

**Fig. 1:**
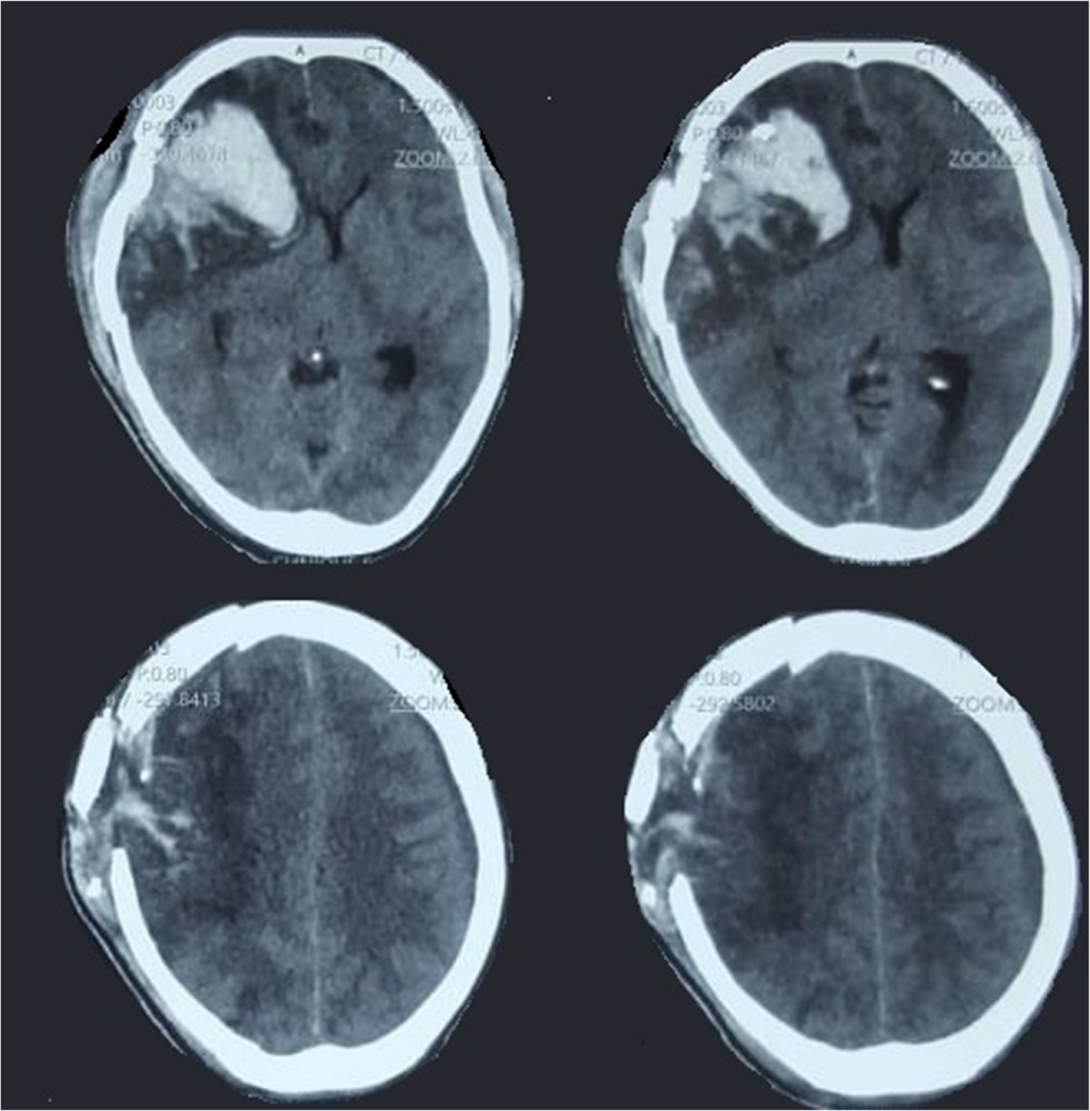
Brain Window CT of a patient with PCCI showing a comminuted right frontal fracture with an underlying frontal burst lobe with severe brain edema

**Fig. 2:**
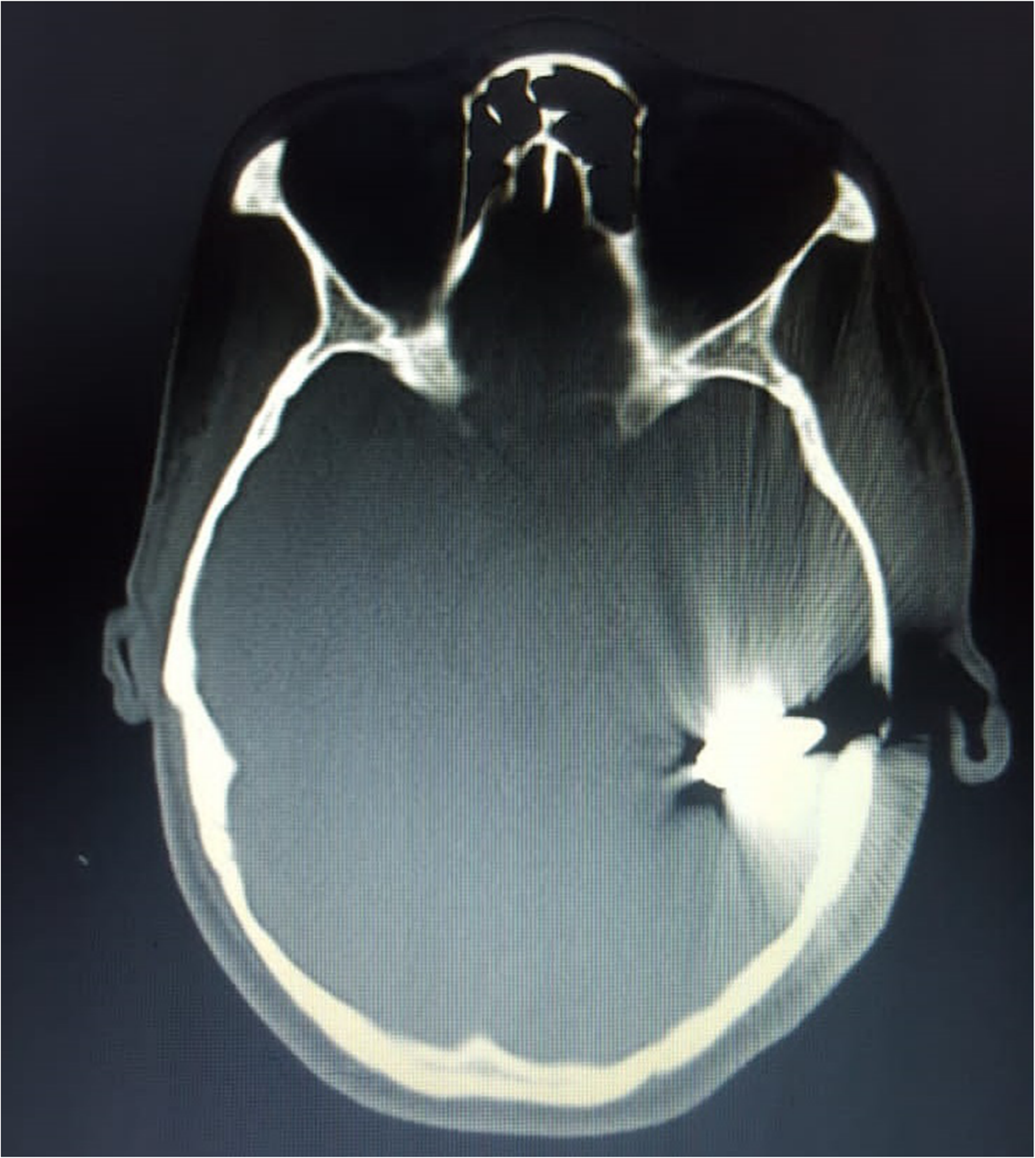
Bone window CT of a patient with PCCI showing a retained bullet in the cranium

About 53.1% of them were admitted within 24 h following the injury, and 82.1% were operated (minor and major surgeries) within the 24 h following the hospital admission. On admission, 40.6% (41/69) were unstable hemodynamically. Three patients had a Glasgow Coma Scale (GCS) < 8, 22 (31.9%) had GCS between 9 to 13, and 44 (63.7%) with a GCS > 13. There were 44.9% of patients who presented with either a focal neurologic deficit at admission, more frequently hemiparesis, generalized tonic-clonic convulsion, and cranial palsies. Forty-four percent of those patients presented with associated injuries (maxillo-facial, musculoskeletal, abdominal, and thoracic, etc.). All patients had skull fractures demonstrated by skull X-rays or a brain tomodensitometry (CT), but only 34 (49.3%) of them had done a brain CT scan, and 8 (11.6%) of them had intracranial bleeding. About 62.3% of patients had a dural tear found during surgery. Figure 1 illustrates an example of a PCCI with intracranial hemorrhage, and Fig. 2 illustrates a retained bullet.

### Management

**Fig. 3:**
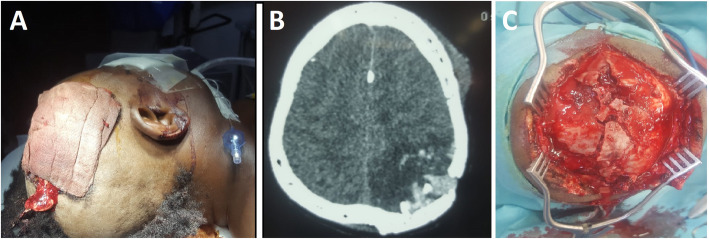
**A**, **B** and **C** illustrates imaging and intraoperative findings of a case of a 46-year old male with PCCI

Medical management administered to those patients was made of prophylactic antibiotics and analgesics (100%), anti-epileptic drugs (62.3%), blood transfusion (18.84%), and osmotherapy (15.9%). The antibiotics used were essentially a combination of Ceftriaxone and metronidazole in most of the cases. Surgical management consisted debridement (100%), duroplasty in 75.4%, craniectomy (71.1%), craniotomy (28.9%), and additional hematoma evacuation. Figure 3 A-C illustrates a case of PCCI with imaging and intraoperative findings. Patients had additional frequent clinical review, and wound dressing in post-operative care.

### Outcomes

Almost half (50.7%) of the operated patients developed post-operative complications, mainly surgical site infections (empyema, brain abscesses, and meningitis) (57.1%) and CSF leakage (3%). Post-traumatic seizures occurred in 6 patients. There were 27 cases of re-operations indicated for abscess drainage (9/27), additional debridement and bone removal (6/27), and scalp skin graft (5/27). The median length of stay in the hospital was 24 days (13 to 43). The overall mortality was 7.2%. In terms of outcomes, 48 patients (69.6%) had favorable outcomes (GOS 4: 23 patients and GOS 5: 25 patients), whereas 25 (30.4%) patients had poor outcomes (GOS 1: 5 patients, GOS 2: 1 patient and GOS 3: 15 patients). Among clinical parameters on admission, patients with GCS < 13 (67% vs 33%, *p* = 0.001), with unstable hemodynamic state (67% vs 33%, *p* = 0.004), with neurologic deficit (90% vs 10%, *p* < 0.001), dural tear (81% vs 19%, *p* = 0.030), and with intracranial hemorrhage (71% vs 29%, *p* = 0.008) had poorer outcomes (Table 1).

**Table 1:** Demographics and clinical parameters of patients operated for PCCI

Variables n (%)		GOS (4–5): *n* = 48 (69.6%)	GOS (1–3): *n* = 21 (30.4%)	Total *n* = 69	*p*-value
Age in years	<=15	16(33%)	5(24%)	21	0.769
	16–45	25(52%)	13(62%)	38	
	46–70	7(15%)	3(14%)	10	
Sex:	Males	36(75%)	16(76%)	52	0.586
	Females	12(25%)	5(24%)	17	
Category:	Civilian	60(95%)	18(86%)	63	0.375
	Military	3(5%)	3(14%)	6	
Injury mechanism:	Gunshot	20(42%)	9(43%)	29	0.927
	Others	28(58%)	12(57%)	40	
Time injury-admission:	< 24 h	24(50%)	13(62%)	37	0.362
	> 24 h	24(50%)	8(38%)	32	
Time injury-surgery:	< 24 h	21(44%)	11(52%)	32	0.508
	> 24 h	27(46%)	10(48%)	37	
Time admission-surgery:	< 24 h	39(81%)	18(86%)	57	0.471
	> 24 h	9(19%)	3(14%)	12	
Hemodynamic instability	Stable	34(71%)	7(33%)	41	0.004
	Unstable	14(29%)	14(67%)	28	
Admission GCS:	> 13	37(77%)	7(33%)	44	0.001
	< 13	11(23%)	14(67%)	25	
Neurological deficit:	Yes	12(25%)	19(90%)	31	< 0.001
	No	36(75%)	2(10%)	38	
*Hemiplegia:*	*Yes*	*2(4%)*	*14(67%)*	*16*	*< 0.001*
	*No*	*46(96%)*	*7(33%)*	*53*	
Types of PCCI:	Dural tear detected	22(46%)	4(19%)	26	0.030
	Tangential	26(54%)	17(81%)	43	
Location of the PCCI:	Unilobar	30(63%)	9(75%)	39	0.130
	Bi or multilobar	18(37%)	12(25%)	30	
	Unihemispheric	44(92%)	20(95%)	64	0.516
	Bi hemispheric and posterior fossa	4(8%)	1(5%)	5	
Intracranial bleeding:	Yes	2(4%)	6(29%)	8	0.008
	No	46(96%)	15(71%)	61	
Depressed skull fractures:	Yes	14(29%)	3(14%)	17	0.155
	No	34(71%)	18(86%)	52	
Associated lesions:	Present	22(46%)	9(43%)	31	0.819
	Absent	26(54%)	12(57%)	38	

### Factors associated with the outcomes of PCCI

When performing univariate analysis between the poor outcomes and all parameters with *p* < 0.05 in chi-square test (and Fischer exact test where appropriate), we found that some factors were statistically associated with poor hospital outcomes: hemodynamic instability (*p* = 0.005 OR = 4.8, CI: 1.61–14.59), GCS < 13(p = 0.001, OR = 6.7, CI: 2.17–20.81), neurological deficit on admission (p < 0.001, OR = 28.5, CI: 5.77–140.70), hemiplegia (p < 0.001, OR = 46, CI: 8.5–247.1), dural tear (*p* = 0.041, OR = 3.59; CI: 1.05–12.28) and intracerebral hemorrhage (*p* = 0.011, OR = 9.2, IC: 1.67–50.51) (Table 2). In multivariate analysis, we found that hemodynamic instability, admission GCS < 13, hemiplegia, and intracerebral hemorrhage were found to be statistically associated with poor outcomes (Table 2).

**Table 2:** Factors associated with poor outcomes: univariate and multivariate regression

	Univariate			Multivariate		
Variables	OR	CI	p	AOR	CI	p
Hemodynamic instability	4.8	1.61–14.59	**0.005**	4.8	1.06–21.5	0.041
GCS < 13	6.7	2.17–20.81	**0.001**	5.3	1.6–18.0	0.006
Neurologic deficit	28.5	5.77–140.7	< 0.001	–	–	–
*Hemiplegia on admission	46	8.5–247.1	**< 0.001**	35	5.4–225.5	< 0.001
Dural tear detected	3.59	1.05–12.28	0.041	–	–	–
Intracranial hemorrhage	9.2	1.67–50.51	**0.011**	7.6	1.1–50.2	0.034

## Discussion

### Demographics and injury factors

The prevalence of PCCI in our study was 9.1% and deemed to be very low, especially due to the survival rate of the victims who reached the facility on time, and the weakness of health-related events reporting systems in a conflict region. Indeed, there are no emergency medical mobile services that can collect and take to the hospital trauma patients to facilities for appropriate care [6]. Most of our patients were young adults, as reported to other literature with an average age of 30 years [8, 12, 13]. In our study, injuries were caused in the majority by gunshot head injury as frequently it is the case in war conflict zones, whereas non-missile low-velocity weapons are mostly reported in another African study [14].

More than half of patients were coming from rural areas, mostly due to the scarcity of the surgical workforce in the Eastern region of the DRC; Kong et al. found that in South Africa more than of half patients with PCCIs were urban, and due to criminalities in the cities [15].

### Clinical presentations and imaging

Patients in our study were admitted with a delay as compared to other studies, but 82.1% were operated in a record time of 24 h following trauma; Aziz et al. reported that the average time from admission to surgery average was 57 h [6]. There were not any differences between these demographic parameters in terms of outcomes as found also by Jamous et al. [9].

On the other hand, Paradot et al. [7], Aarabi et al. [5], and Wei et al. [16] found respectively that the age (< 15 years vs > 16 years) of patients, the time between injury and admission (< 8 h vs > 8 h) were differently distributed in terms of outcomes. This may be explained by the difference of distribution of age and time from injury to the hospital between our study and theirs [5, 16]. Literature has documented that patients victims of PCCI had most of the time a low GCS and unstable hemodynamic state [4, 17, 18]. But in LMICs, different results are found because patients with low GCS die at the injury place or before admission. An important proportion of them was admitted with hemodynamic instability and neurologic deficit (especially hemiplegia in 23.6%) in our study. As found in our study and as already found in previous ones, patients with hemodynamic instability and low GCS had poorer outcomes than patients without these characteristics [8, 12, 19, 20]. In our study, criteria found in the ATLS® protocol 9th edition were used to classify patients as hemodynamically stable or unstable, but mainly the systolic blood pressure and the level of consciousness were consistently captured. Only half of the patients had done a Brain CT scan because of a lack of financial resources and poor clinical status at admission as compared to the study done by Wakrim et al. in which 100% of patients were diagnosed by a Brain CT scan [21]. Eight patients had an intracerebral hemorrhage and they had poorer outcomes than patients who had not such lesion contrarily to Petridis et al. who found did not find the same results [22]. Such findings can be explained by the fact that in our study half of the patients did not have brain CT scanning. Other important factors such as pupil response to light and midline shift in the brain CT were found in other studies to be differently distributed [18, 22] but were not looked at in our study due to the inconstant medical records.

### Management and outcomes

The medical treatment consists of antibiotics and anti-comitial treatment to which we can add other treatments according to the patient’s state. That is why in our series all patients were treated with antibiotics and analgesics but only 62.3% were treated with anti-epileptic drugs for seizure treatment or prophylaxis like done by Thiam et al. [14]. This rate of duroplasty is high compared to the rate found by Thiam et al. and it may be explained by the high number of penetrating wounds in need of such procedures. The length of hospital stay in our study was longer as compared to other studies [12, 23]. It may be explained by the high number of postoperative complications and re-operation dominated by infections in most cases as found in other studies [24, 25]. Mortality and poor outcomes were observed to be lower in our study as compared to others. This may be a masked feature of the high mortality of patients who die within the golden hours before reaching the hospital due to the lack of emergency medical mobile services [6, 7, 14]. In HICs with a well-organized Health System, such emergency services allow most of the victims with PCCI to reach the specialized hospital for appropriate management. This fact seems to reveal the high rate of real-life in-hospital mortality and poor outcomes related to PCCI [17, 23, 26].

### Factors associated with outcomes

on univariate analysis, poor outcomes were found, as in other studies, among patients with low admission GCS score [3, 5, 12] and with the presence of intracranial hemorrhage [5]. However, Khan et al. [12] did not find that dural tear in PCCI to be associated with poorer outcomes as found in our study. Many other factors have been described in different studies to be associated with poor outcomes on univariate analysis: bilateral mydriasis, bi-hemispheric lesion [12, 23, 27, 28], trans-ventricular penetrating agent trajectory, high intracranial pressure, [3], cistern obliteration [5] but not found in our study because they were not evaluated on admission and a low rate of accessibility to brain CT scan. Hemodynamic instability is well-known to be associated with poor outcomes in trauma patients as reported in our study [23]. Four factors were associated with poorer outcomes in the multivariate logistic regression model: GCS < 13, hemodynamic instability, intracranial hemorrhage, and hemiplegia. Our finding comforts those of Gressot et al. [3] in terms of GCS; he found that having a GCS between 9 and 15 on admission was statistically associated with favorable outcomes. However, other factors associated with poor outcomes in a multivariate regression model have been found (patency of basal cisterns, nonreactive pupil reaction, and midline shift) by Aarabi et al. whereas Gressot et al. found that younger age and uni-hemispheric or bi-frontal lesion are associated with favorable outcomes [3, 5]. These findings are emphasized by a literature review on PCCIs done by Aarabi et al. in 2015 based on old studies and his personal experience [29].

### Limitations

Our study had several limitations, as per nature to be a retrospective series. The dynamics of the surgical workforce and skills over 10 years period could have contributed to the surgical outcomes. There was a relatively big number of patients with PCCI who unluckily did not reach the tertiary hospital alive or who died at the injury site. The low number of our cases does not allow us to extrapolate our findings in case they are different from pre-existing ones in the literature. Some important clinical factors (pupil response to light, intracranial pressure, midline shift in the brain CT scan, and type of intracranial hemorrhage) which could probably be associated with poor outcomes were not routinely documented on the medical records. The other limitation of this study is the fact that some of the patients from our series have done only from head X-ray examination because of financial accessibility, several breakdowns of the CT scan services in the hospital during the study period; this could have unveiled other intracranial lesions associated to poor prognosis. In addition, our setting does not perform advanced ICP monitoring and relies mainly on clinical findings to monitor patients.

## Conclusion

The factors associated with poor hospital outcomes of patients with PCCI in armed conflicts region are hemodynamic instability, admission GCS < 13, the presence of intracranial hemorrhage, and hemiplegia. In the postoperative period, patients with PCCI frequently developed complications, especially infectious. The poor outcomes may be improved by strengthening the local capacity in the acute care management of trauma patients. There is a need for optimizing the initial care of all trauma patients by rigorous trauma protocol starting from the injury site to the hospitals, especially in armed conflict regions. Further studies are advocated to find out the outcome predictors in the long-term follow-up of patients with PCCIs.

## Data Availability

The datasets generated during and analyzed during the current study are not publicly available due to legal and ethical reasons but are available from the corresponding author on reasonable request.

## Abbreviations

AOR: Adjusted Odds ratio
GCS: Glasgow coma scale
GOS: Glasgow outcomes score
HIC: High-income countries
LMIC: Low and middle-income countries
PCCI: Penetrating craniocerebral injury
OR: Odd Ratio
CI: Confidence interval
